# Challenges in Managing Acute Cardiovascular Diseases and Follow Up Care in Rural Areas: A Narrative Review

**DOI:** 10.3390/ijerph16245126

**Published:** 2019-12-15

**Authors:** Sandra C. Thompson, Lee Nedkoff, Judith Katzenellenbogen, Mohammad Akhtar Hussain, Frank Sanfilippo

**Affiliations:** 1 Western Australian Centre for Rural Health, The University of Western Australia, P.O. Box 109, Geraldton 6531, Australia; drakhtarhussain@gmail.com; 2 School of Population and Global Health, The University of Western Australia, M431, 35 Stirling Highway, Perth 6009, Australia; lee.nedkoff@uwa.edu.au (L.N.); Judith.katzenellenbogen@uwa.edu.au (J.K.); frank.sanfilippo@uwa.edu.au (F.S.); 3 Menzies Institute for Medical Research, University of Tasmania, 15-17 Liverpool Street, Hobart, Tasmania 7000, Australia

**Keywords:** rural, remote, acute coronary syndrome, revascularization, cardiac rehabilitation, cardiovascular, prevention, workforce, primary care, access, outcomes, review

## Abstract

This narrative review explores relevant literature that is related to the challenges in implementing evidence-based management for clinicians in rural and remote areas, while primarily focussing on management of acute coronary syndrome (ACS) and follow up care. A targeted literature search around rural/urban differences in the management of ACS, cardiovascular disease, and cardiac rehabilitation identified multiple issues that are related to access, including the ability to pay, transport and geographic distances, delays in patients seeking care, access to diagnostic testing, and timely treatment in an appropriate facility. Workforce shortages or lack of ready access to relevant expertise, cultural differences, and complexity that arises from comorbidities and from geographical isolation amplified diagnostic challenges. Given the urgency in management of ACS, rural clinicians must act quickly to achieve optimal patient outcomes. New technologies and quality improvement approaches enable better access to rapid diagnosis, as well as specialist input and care. Achieving an uptake of cardiac rehabilitation in rural and remote settings poses challenges that may reduce with the use of alternative models to centre-based rehabilitation and use of modern technologies. Expediting improvement in cardiovascular outcomes and reducing rural disparities requires system changes and that clinicians embrace attention to prevention, emergency management, and follow up care in rural contexts.

## 1. Introduction

Cardiovascular disease (CVD) is a collective term for diseases of the heart and blood vessels. It is a major cause of morbidity and death globally, and it is a major cause of widening inequity in health outcomes between the rich and poor [1,2]. In high-income countries, there has been a remarkable reduction in mortality from CVD over the last few decades, although CVD remains a leading cause of death [3,4]. Reduced mortality rates are attributed to multiple factors, which include public health prevention (for example, through improved nutrition and a reduction in smoking rates), medical management (such as for hypertension and dyslipidaemias through the development and uptake of highly effective pharmacotherapies), and procedural interventions that involve coronary revascularisation (coronary artery bypass grafting or stents).

Rural clinicians/hospitals must recognise the critical importance of minimising cardiac ischaemia and the risk of potentially fatal arrhythmias in managing acute cardiovascular events while the prevention and medical management of established cardiovascular risk factors or disease can be delivered in rural settings. Increasingly, it has been recognised that minimising the time of cardiac muscle ischaemia reduces the damage to heart muscle and, therefore, the onset of long-term sequelae, such as poor ejection fraction and heart failure.

This review fits the typology of a narrative review, which provides a broad summation of a topic area of value for those coming to a subject for the first time. It explores the general challenges of treating CVD in rural settings in high-income countries. However, understanding the facilitators and barriers to CVD prevention and management in countries that are well resourced can also inform the deployment of strategies that have proven to be effective in low and middle-income countries.

When referring to rural areas, we consider geographic areas that are located outside of metropolitan cities and that typically have a low population density. Approaches to classifying rural areas will differ by country, for example, Australia uses the term “rural and remote” for areas outside of Australia’s Major cities, classifying areas as Inner regional, Outer regional, Remote, or Very remote [5]. However, all of the countries will have increased challenges delivering the type of care needed for managing acute CVD as remoteness increases. Countries will differ between rural and urban areas with respect to the disparity that exists between disease incidence and in access to appropriate primary, medical, and surgical care for CVD. Indeed, large disparities exist between rural and remote areas within large countries. There are also likely to be gaps in the availability and coverage of health data in rural and remote areas, and in information available at the local area level.

## 2. Methods

A narrative review provides an up-to-date overview of issues and evidence-round up on specific health care topic, and it has advantages and disadvantages in not following the approach of systematic evidence-based reviews [6,7]. We focussed on issues that were related to prevention and the management of coronary heart disease, particularly related to acute coronary syndromes (ACS), in rural areas of high-income countries. The authors’ knowledge and understanding of cardiovascular disease, management of ACS, and rural health and cardiovascular disparities was supplemented by a targeted literature searches while using PubMed and Google Scholar, and focussed on rural/urban differences in aspects of prevention, diagnosis, and management of ACS/CVD and rural secondary prevention and cardiac rehabilitation. Therefore, the review draws heavily upon literature from Australia, where the authors have a good understanding of the context, challenges, and literature relevant to rural urban differences for CVD prevention and treatment. However, we also reference some relevant findings from other high-income countries, as these issues are not unique to Australia.

## 3. Burden of CVD in Rural Areas

The health of a population can be measured in many different ways while using indicators that reflect mortality, morbidity, overall well-being, lifestyle behaviours, and other health-related risk factors [8]. However, across many health conditions, there is a health disadvantage in rural areas and this is also true for cardiovascular diseases. In the United States, Eberhard and colleagues reported that the heart disease mortality rate was the highest in the South and 25% higher in rural areas as compared to the rate among Southern suburban residents [8]. Other studies show similar inequities with persistently higher coronary heart disease rates in rural Appalachia, despite overall reductions in CVD [9] and a large excess of chronic heart disease (CHD) rates in female residents who lived in rural counties [10]. Australians living in rural and remote Australia experience more CVD risk factors, higher rates of CVD-related hospitalisation, and are more likely to die of CVD than those in the metropolitan areas. The further that a person lives from a metropolitan centre, the greater their risk of hospitalisation and death from cardiovascular disease [11]. In general, the burden of CVD is felt more by lower socioeconomic groups, Indigenous people, people from diverse cultural backgrounds, and those living in rural and remote communities. However, it is worth highlighting that rurality, gender, and race interact in a complex manner, reflecting impacts on both incidence of disease and access to care for those who have a cardiac illness. For example, analysis undertaken in Western Australia highlighted the disproportionate incidence of myocardial infarction (MI) rates in Indigenous people particularly occurring at younger ages and in women [12]. However, this study also reported that geographical disadvantage is not consistently associated with a greater burden of disease, with an interplay of many factors that contribute to the complex relationship between MI incidence and sex, age, Indigenous status, and residence [13]. A study that was conducted in New South Wales (NSW) also identified the importance of contextual influences on disparities in MI rates, and in mortality and procedures after the admission for acute myocardial infarction (AMI) [14]. Area-level socio-economic and rural disadvantage could partly explain the significant variation in AMI rates by area [15].

## 4. Management of CVD

### 4.1. Prevention

A reduction of identified risk factors is needed in order to prevent or manage CVD. Traditional risk factor management focusses on the following factors for both primary and secondary prevention: smoking cessation, control of hypertension and hypercholesterolemia, good nutrition, reduction of obesity, and being physically active. There is now an increasing understanding that behavioural and biomedical approaches to prevention are inadequate and that the social determinants of health underpin the ability of disadvantaged people to follow advice regarding risk factor management [16]. Increasingly, social and historical factors are recognised as influencing the health and uptake of medical advice and treatments.

People in rural and remote areas might experience more problems in accessing and following advice that is relevant to prevention [17], and often have lower educational attainment, poorer health literacy, experience more food insecurity, and have poorer or a higher cost to access healthy foods [18,19,20]. The rates of smoking are higher in rural areas and in people experiencing stress or mental illness [21,22], and the use of services that support quitting smoking has been found to be lower in rural residents [23]. There is a known higher prevalence of risk factors, such as rates of alcohol consumption and obesity in rural Australia [24]. Alston and colleagues have reported that one-third of the excess CVD deaths in rural residents were due to risk factors, and that approximately 1461 deaths from CVD could be delayed annually, reducing the rural-urban mortality gap by 38.2% if rural Australian residents had the same levels of risk factors as those in metropolitan areas [25].

Rural areas are often areas of health workforce shortage, and they have reduced health infrastructure and higher costs of health care delivery [26]. On average, rural patients visit their GP 1–2 fewer times per year than other Australians [27]. Furthermore, research suggests that, throughout Australia, there is substantial under-treatment by GPs of patients with a high risk of CVD [28]. Rural patients get fewer prescriptions for critical medications used in the treatment of CVD, such as beta blockers, ACE inhibitors, statins, and warfarin than other Australians, despite similar or a higher burden of disease than in metropolitan areas [29]. However, complex interactions with ethnicity and income also influence health-related risk behaviours relevant to CVD prevention [30].

### 4.2. Importance of Delays in Acute Coronary Syndrome

It is important to reflect on the evolving state of knowledge when discussing acute cardiac presentations in rural areas. Delays in reaching hospital are important, as arrhythmias can occur at any time. Sudden cardiac death following a MI occurs through ventricular fibrillation (VF), an arrhythmia in which the heart rhythm is rapid and erratic and it fails to pump blood around the body, which results in the rapid loss of consciousness and death. Ongoing ischaemia of cardiac muscle as a consequence of blockages in coronary arteries will result in compromised cardiac function and output. Coronary reperfusion with thrombolysis or angioplasty within the first 12 h can halve the death rate, but the benefit rapidly declines with treatment delays [31].

Delays occur in patients reaching the rural health facility, and this might be because of difficulties with transport, both to the initial health service and then for retrieval in specialised transport to the service with adequate facilities for imaging and revascularisation procedures. Action has been taken within health settings to expedite the treatments that will save heart muscle by expediting transfer to settings in which definitive interventions can occur to maximise the reperfusion of damaged myocardium. However, delays that occur before patients present for medical attention are much more difficult to address.

Multiple reasons contribute to patient delays, including: poor symptom recognition, often amidst co-morbidities; lack of significance being attributed to symptoms in the face of other demands and life challenges; responsibilities that are associated with gender roles; sociocultural priorities; cultural/communication disjunction with health services; and, implications of symptom response. In rural areas, there are additional challenges that are associated with the time to actually reach the medical facility and scarcity or the lack of readily available and suitable transport options. The distance in remote areas presents formidable challenges in reducing delays and climatic conditions can also impact it [32,33].

Efforts to reduce patient delay have included education and mass media strategies to increase public awareness. However, while these efforts may improve the recognition of heart attack symptoms in patients, they have failed to shorten pre-hospital delays. Hence, it is now recommended that future campaigns should reduce the psychosocial and behavioural blocks to action and focus on people at highest risk, including those in rural locations [31].

In more remote areas, a patient’s first point of health care might be a health facility that does not have a doctor routinely present, so rapid assessment and triage is important, with transfer by road or air in an ambulance with a health worker escort generally being needed. Nevertheless, rural hospitals, even with experienced doctors, usually lack the facilities for coronary artery revascularisation procedures. After stabilisation, patients may be transferred to larger urban centres, where cardiac catheterisation can be undertaken for diagnostic imaging of coronary arteries and subsequent revascularisation procedures. Adherence to clinical guidelines and suggested diagnostic and treatment pathways within hospitals can improve the patient outcomes; hence, jurisdictions in the developed world are likely to have ACS models of care (guidelines) to ensure that the treatment and referral pathways are appropriate and that they address local circumstances and treatment availability [34].

Although cardiac arrest is eminently treatable by cardiopulmonary resuscitation (CPR) and defibrillation, this requires access to people appropriately trained and rapid access to a defibrillator. The concept of the Chain of Survival [35] was developed over 30 years ago to highlight that there is a sequence of interdependent time-critical interventions in which bystanders have a critical role in initiating the call for an ambulance, providing life-saving first aid (CPR), and where possible, while using an automated external defibrillator (AED), when cardiac arrest occurs outside of hospital [35]. Prompt action by bystanders can help to ‘buy time’ until more definitive treatment is available by the ambulance paramedics, and bystander CPR in this period more than doubles the chance of survival [36,37], with differences in the rates, interventions, and survival in different regions [38]. The training of takers of emergency ambulance calls also has the potential to improve bystander contributions to survival [39].

### 4.3. Challenges with Diagnosis-Assessment and Management of Patients with Acute Cardiovascular Symptoms

Another component of delay in diagnosis can be the difficulties for clinicians in making a diagnosis; lack of suitability of specialist-developed guidelines for acute rural episodes; difficulties accessing specialist advice; and, a mismatch between the patient transport rules that may not fund the travel of a family member and meet the support needs of the patient. While evidence-based guidelines for assessment and management do exist, adherence to the optimal pathway might not occur in rural hospitals [34,40].

Health providers can be challenged by non-classical or atypical presentations that may occur in patients with complex co-morbidities or for whom the cultural expression of symptoms and pain might differ. The presence of language differences might compound these assessment difficulties. For example, in remote Australia, many Indigenous people do not have English as their first language and they may be assessed by an overseas trained doctor who also does not have English as a first language [41]. Moreover, the cultural expression of pain might differ, with Indigenous men in particular said to minimise the expression of pain, as befits a “tough old bushman” [42]. Rural patients may feel particularly vulnerable, especially if they have little education and family or peer support, and fear being transported to a less familiar environment [43].

Any presentation with acute chest pain or other symptoms, such as jaw or arm discomfort or breathlessness, should be rapidly assessed in the nearest hospital or health centre given the potential for serious and fatal consequences resulting from ACS. Modern assessment includes a 12-lead electrocardiograph (ECG) and blood tests for troponin. The primary aim of early assessment of suspected ACS patients is accurate risk stratification, thus ensuring that the patient is managed while using an appropriate diagnostic and management pathway. It is now possible for less experienced care providers in rural areas to upload ECG traces to larger centres, where they can be rapidly read and interpreted by those with substantial experience [44], but how widespread availability is in practice is unclear. When cardiac troponin was initially introduced in Australia as the main diagnostic biomarker for MI, it was only available in metropolitan laboratories, but, over time, became available in most rural hospitals. The penetration of the test into regional settings has concurrently occurred with greater sensitivity and, more recently, the development of point-of-care troponin testing [45]. However, lower specificity accompanies the increasing sensitivity of such tests results [46], so a clear understanding is required of the need for serial troponins in conjunction with interpretable ECGs for the accurate diagnosis of AMI. Point-of-care testing for troponins has made the rapid assessment of patients in remote areas easier, with the accuracy of these tests so improved in recent years that even single test troponin has comparable discrimination ability to a high sensitivity cardiac troponin assay for ruling out AMI after a single blood test [47].

Emergency reperfusion is recommended by clinical guidelines in developed countries for patients with an ST-elevation myocardial infarction (STEMI) without contraindications and who present within 12 h of symptom onset [48,49,50]. For those in metropolitan settings, this is likely to be primary percutaneous coronary intervention (PCI), a procedure where the narrowed or blocked coronary arteries are opened, most commonly with a small, wire mesh tube (stent). In this acute setting, it should be performed within 90 min of first medical contact. For rural patients, the time that is required for emergency retrieval to a centre with procedural revascularisation capability usually exceeds 90 min, and therefore medical treatment with fibrinolytic therapy is implemented instead. Transfer of patients treated with fibrinolytic therapy to a facility with cardiac catheterisation and PCI-capability if required is recommended to occur within 24 h. It is also recommended that angiography for coronary revascularisation is used to assess high and very high risk patients with non-STEMI ACS [51], so their transfer to a suitable facility for this is also indicated.

It is recommended that patients with diagnosed ACS be monitored, given that death is most likely to occur as a result of arrhythmias. In large metropolitan centres, this would normally be in a setting, such as a coronary care unit. In rural settings, less specialised monitoring might be all that is available until suitable transfer to a larger centre occurs. The medical management of acute ACS might also include other medications, such as aspirin, platelet P2Y12 receptor antagonists (such as clopidogrel, ticagrelor, and prasugrel), beta blockers, and other treatments for those at high risk of ischaemic events, depending on risk of thrombotic or bleeding events. Further discussion of such treatments is beyond the scope of this review.

### 4.4. Lack of Access to Surgical and Catheterisation Facilities in Rural Areas

The facilities available in regional and rural areas vary enormously and they will largely reflect the population numbers in the regional centre and the transport routes that serve the populations in surrounding areas. However, procedural management of ACS relies upon access to cardiac catheterisation facilities and specialist services which are often not available in rural areas. This can potentially impact on optimal timely management for ACS patients. A study from New South Wales, Australia, demonstrated that MI patients who were transferred, often from regional to metropolitan hospitals with catheterisation facilities, had better long-term survival than patients who were not transferred, independent of their risk profile [52], which indicated the importance of safe, timely transport to centres where specialist services and facilities exist.

Surgical intervention for patients with coronary heart disease includes coronary artery bypass grafting (CABG), an operation where a blood vessel taken from elsewhere in the patient’s body (chest, leg or forearm) is grafted (attached) to a person’s coronary artery to let blood ‘detour’ past a narrowing or blockage in their artery. This is less commonly undertaken for acute patients; however, CABG is a necessary revascularisation option for some acute and stable CVD patients, particularly those with multi-vessel disease or at higher risk, such as people with diabetes [53,54]. There are recovery issues following CABG, including wound care and restrictions on driving or flying, which have implications for patients from rural areas with respect to daily activities and medical clearances. Other concerns, such as prevention of deep vein thrombosis requiring compression stockings, access to adequate supply of regular medications for secondary prevention, and timely access to follow-up care, all impact patient recovery and discharge.

### 4.5. Cardiac Rehabilitation and Availability of Services for Secondary Prevention

Patients who survive will generally be required to make lifestyle modifications to reduce their chance of recurrence and rehospitalisation, and optimise their quality of life, regardless of what treatment was needed at the time of their MI. There is good evidence that secondary prevention and cardiac rehabilitation (CR) can make a substantial difference in reducing ACS recurrence, hospital readmission, and mortality [55]. Medical management of risk factors is particularly important for this, with general practitioners (GPs) having a key role. However, GPs are more likely to prescribe ACS medications than to assist in lifestyle or psychological management, so the referral to CR programs which offer comprehensive lifestyle interventions remains important. Data are limited on interventions that are effective in improving general practitioner management in rural, minority, and Indigenous populations [56].

CR is a well-established approach for increasing cardiovascular fitness through exercise programs that have been recommended [57]. Typically, exercise-based CR aims to achieve 20–60 min. of moderate intensity continuous exercise, 3–5 times a week, and includes muscular strength and endurance exercises. Modifying CVD risk factors, improving the quality of life, and enhancing medication adherence through education and psychological and social support are also the objectives of CR [55]. Providing access to healthy lifestyle options has traditionally not only included exercise programs, but has occurred through education to improve the understanding of the reason for medications, cooking demonstrations, and health and peer support as drivers for behavioural change.

The nature of the programs has meant that CR program delivery has traditionally been centre-based. Uptake has been suboptimal, despite evidence for the effectiveness of CR, uptake, with lesser uptake for residents located at a distance from a CR centre, and even more so for those living in rural areas [58]. These challenges increase for those living outside of major rural centres, where regional hospitals employ a wider range of health professionals who can contribute to cardiac rehabilitation programs. Efforts to link a patient to primary care and rural CR after discharge from a specialist (often metropolitan) facility following an acute event are important in uptake, and they include the medical team stressing the importance of CR and arranging the initial referral/appointment [59]. The critical need for improvements in communication around discharge, better integration with primary care, and attention to the special needs of vulnerable populations has been well described [60,61].

Alternatives to centre-based CR programs have been trialled and shown to result in equivalent benefits [62,63,64], although different approaches may present facilitators or barriers to particular people. A systematic review of the alternative models of CR recommended that, for rural and remote populations and harder to reach groups, local healthcare systems should strive to integrate alternative models of CR, such as brief telehealth interventions that are tailored to individual’s risk factor profile, as well as community- or home-based programs, as having choices can help to ensure that programs fit patients’ needs, risk factor profile, and preferences [57,65,66,67]. The challenges of access for rural and remote patients are regularly referred to as reasons for utilising a wider range of options for how CR can be delivered and the use of virtual or remote support through telephones, the use of smartphone apps and internet programs is an area of ongoing development and research [68,69].

The landscape with respect to CR is changing for clinicians who manage patients in rural and remote areas following an acute coronary event. A recent meta-analysis of exercise-based CR programs cast doubt on the benefits of exercise-based CR programs for reducing cardiac mortality [70]. A study that was conducted in NSW also identified the importance of contextual influences on disparities in MI rates, and in mortality and procedures after admission for AMI. The significant variation in AMI rates by area could be partly explained by area-level socio-economic and rural disadvantage [15], which might reflect a greater impact of improved CVD diagnosis and treatment, or inadequacies in measuring exercise participation [71]. Abell and colleagues note that exercise participation at sufficient intensity, frequency, and duration is proposed to be the key mechanism for improvements in cardiorespiratory fitness and, thus, reductions in mortality and morbidity [71]. These are key messages for rural clinicians to emphasise in managing patients with heart disease—reiterating the importance of reducing risk factors, adherence to evidence-based medications, and sufficient exercise to improve cardiovascular fitness. Approaches that support key aspects of CR are likely to become more sophisticated, allowing for providers access to information on their patient’s participation in, and adherence to, CR programs. However, not all patients will manage remote devices or be keen to allow smart monitoring, although the interest of the clinician in the person’s progress and ongoing encouragement will remain important.

### 4.6. Differences in Patient Populations

It is well known that patients living in rural and remote areas have poorer access to health services. However, multiple dimensions of access to services have been described, with distance only comprising one aspect of this. Levesque and colleagues described a conceptualisation of access to health care that included the opportunity to identify healthcare needs, to seek healthcare services, to reach, to obtain/use health care services, and to actually have a need for services fulfilled [72]. These authors conceptualised five dimensions of accessibility: approachability; acceptability; availability and accommodation; affordability; and, appropriateness [72]. Based upon this framework, they identified five corresponding abilities of populations that interact with the dimensions of accessibility to generate access: ability to perceive; ability to seek; ability to reach; ability to pay; and, ability to engage.

This framework underscores particular barriers that are more likely to occur in rural and remote populations and that make the management of heart disease for rural clinicians more challenging. The first is health literacy issues that include the education level of the patient/population, their ability to recognise and respond appropriately to symptoms, and the knowledge and awareness of others around them. The ability to pay is another critical issue for some populations, and there are documented different uptakes of private health insurance, which, in turn, results in differential access to health care in a specialist health facility, differential cardiac angiography, and revascularisation rates [73].

There are particular population groups who have poorer outcomes. In Australia, attention is now focussed upon Indigenous people where CVD accounts for a substantial proportion of their life expectancy gap with the general population [32]. The reasons for this are complex and multifactorial, and they have been well described elsewhere [33,74]. The fact that Indigenous people represent a greater proportion of the population as remoteness increases is one contributing factor. Difficulties for clinicians and services arise from the many patient and service-related factors that align to add to the difficulties of timely presentation, assessment of symptoms, cultural and language challenges, willingness to take up treatment and adherence to recommended advice and medication, and clinical complexity. Worse outcomes attributed to rural hospitals may be insignificant or show a pattern of outcomes that is less easy to explain simply on the basis of rural location once these multiple complex factors that are associated with particular patient populations and clinical findings are taken into account in multivariate analyses [13,75].

Two other issues impact disease management in rural areas. The first relates to multiple comorbidities that may mask or confound the diagnosis of acute CVD, and also affect ongoing disease management [34,76,77,78,79]. The second is gender differences in the epidemiology of CVD and also how women present with acute CVD. Cardiovascular disease was considered to be a “male” disease for a long period of time due to higher absolute rates of MI and CHD in men when compared with women, although the relative risk of CVD morbidity and mortality is actually higher in women. The gender differences in symptom presentation and CVD risk in women is increased, to a greater extent, by some traditional factors (such as diabetes, hypertension, hypercholesterolemia, and obesity) with socioeconomic and psychosocial factors also have a higher impact on CVD in women [80]. There were well described biases in favour of men in terms of accurate diagnosis and active management late last century, including transfer to appropriate facilities and less use of interventions that were thought to contribute to a poorer outcome in women [81,82,83,84]. There continues to be evidence that women are likely to be managed more conservatively and with less intervention than men, despite underlying differences in the disease processes that account for some of the differences in how males and females are treated [85,86,87]. This could be due, in part, to aetiologic differences in ACS between women and men, with Type 2 MI (caused by imbalance between oxygen demand and supply, such as microvascular dysfunction) being more common in women than men [88]. Furthermore, Type 2 MI is more difficult to diagnose than the more common Type 1 MI, which includes STEMI and non-STEMI [89]. Rural clinicians and hospitals need to be aware of the evolving understanding of gender differences in CVD that are relevant for prevention as well as management [90,91].

## 5. Workforce Challenges

Their greater challenge with recruitment and retention of the health workforce is a critical issue facing many rural and remote areas [92]. Solving the maldistribution of the health workforce is an issue for both developing and developed countries, and it has proved to be a “wicked” problem, being relatively recalcitrant to interventions [93]. A consequence is not only workforce shortages, but also workforce turnover, which in itself results in lower levels of experience in health/medicine and understanding of the local context and poorer continuity of care. Nurses are a key component of the health workforce, and particularly so in more remote areas, and they have an important role in many aspects of prevention and management of CVD [94,95,96,97,98]. Pharmacists, allied health professionals, and Indigenous health professionals also make important contributions, including to systems change to better support the needs of patients [32,42,61,99,100,101].

There has been a lack of or relatively poor access to specialist care for rural patients with CVD [102,103], and, of course, it is not possible to have local specialists where there is an insufficient clinical case load for a doctor to build and maintain the relevant clinical skills. For these reasons, regional centres will not have resident specialist expertise until they reach a sufficient size to justify basing such a resource there. Having the relevant facilities to enable them to utilise their full scope of practice and to maintain annual volume recommendations for procedural skill maintenance are other important considerations, as is the need for support from other highly trained members of the multidisciplinary care team.

Workforce shortage has other flow-on effects in terms of expertise to support the education and training of other practitioners and assist with keeping them up to date with emerging technologies and evidence around the best practice treatment [104]. Access to continuing education via locally based programs can help in ensuring that rural practitioners do not feel professionally isolated, and it is likely to be undertaken in rural settings in a way that encourages interprofessional input and can encourage retention [105]. Access to high quality information via the internet and use of webinars has changed the landscape for professional development [106,107,108]. Such connectivity is generally readily available in developed countries [107,108], although attendance and keeping up to date may be difficult for time-poor rural practitioners. Distance supervision and professional support are also useful in training for rural practitioners [108], although there are few reports of initiatives that are specific to cardiovascular care.

Commonly, the management of acute CVD incorporates care based upon clinical care pathways that incorporate decisions based on clinical and investigatory findings. Clinicians do not always deliver the care that they think they deliver or mean to deliver. This emphasises the importance of looking at what is in fact delivered for aspects of care. Audits of clinical practice and outcomes within rural hospitals with feedback to clinicians and management can be a powerful means of engaging them in care and systems improvement [34,109]. General practitioners and their practice team have an important role in improving patient management, given the importance of primary health care following an acute coronary event, with education and integration of care with hospitals offering particular benefit for improving evidence based treatments [110]. Peer support programs can help to build patient skills in chronic disease self-management and provide social support, particularly while using a combination of face-to-face and virtual meetings [111].

## 6. Challenges in Quality of Care in Rural Hospitals

Suboptimal health care occurs in both urban and rural hospitals. This can come to attention because of publicised disasters or as a result of systematic assessment of performance and variation between hospitals. Considerable attention has focussed on disparities in care between rural and urban hospitals in the US, while recognising that smaller rural hospitals often have fewer resources and less funding than larger urban hospitals, following the Institute of Medicine reports into the quality of health care in the United States [40,112]. Acute MI and heart failure are among the most common clinical conditions and so are often considered in explorations of disparities in care between urban acute care and rural critical access hospitals. Rural hospitals generally perform more poorly in terms of readmission rates, although there has been caution recommended regarding how any penalties for poor performance are applied [113]. The reasons for apparent disparities may include deviation from evidence-based recommendations for management in the acute phase and deficiencies in care at discharge and follow up. However, there is also evidence that differences in care are not only based on clinical assessment of need [34,48,114,115], reinforcing the need for well-developed care pathways and monitoring practice standards to encourage change in systems of care and clinician behaviour [116]. While efforts at highlighting the disparities and quality improvement of care continue, particularly for hospitals that serve those with the greatest health disparities [32], others have argued that having a national standard for health care quality is not an attainable goal. This argument reflects the different spectrum and content of care provided in large cities and that quality standards need to be practical, useful, and affordable for where they are to be implemented [117].

## 7. Opportunities to Overcome Problems Associated with Managing CVD in Rural Areas

Each component of the prevention and treatment pathway comes with its own challenges and it will be difficult to overcome the disparities in outcomes following an acute coronary event for patients living in rural areas when compared to their urban counterparts. Improved communication and integration of care are recurring themes that are identified for improving care [60,118,119]. These largely rely upon clinicians ensuring that the systems of care support individual patients across primary, secondary, and tertiary care interfaces, while recognising that the typical rural patient will experience more care interfaces than those that are located in an urban setting, often with less social support around them when their care is discussed. Efforts must embrace approaches to ensure that primary care providers are aware of what has been happening with their patients and that they understand and agree with the proposed management plan. This will facilitate the consistency of information and care support. The role of different health professionals in contributing to patient care and support is also acknowledged and this is particularly the case in rural areas, with doctors, nurses, physiotherapists, dieticians, diabetes educators, pharmacists, and others all contributing to multidisciplinary team care in management and support. Supporting health education in non-clinical settings, such as community centres, can improve understanding, access to social support, prevention, and treatment [99,120,121].

A recent systematic review of clinical trials investigating the effectiveness of interventions to improve the cardiovascular healthcare in rural areas identified eighteen studies that were all conducted in high-income countries [122]. The five studies involving interventions targeting MI were based on organisational changes, such as those mentioned above and aimed to reduce the time to treatment and decrease mortality. The interventions evaluated included mobile coronary care units, introduction of a new emergency department in a rural hospital, a new protocol for immediate/urgent transfer of patients for PCI, and the implementation of an AMI clinical pathway incorporating thrombolytic administration in rural emergency departments. While educational interventions improve knowledge and self-care, and organisational interventions can improve healthcare processes, their impact on mortality, and other important health outcomes still remains to be established [122].

There is increasing understanding and awareness of the factors that underpin poorer outcomes, with investment in a number of emerging technologies to address some of the challenges that occur for patients and treating clinicians. Telehealth and mHealth both offer opportunities for using expertise located elsewhere to help with the diagnosis and management of a condition. The technology must function well and be user friendly to work as an accepted method, being beneficial for both the patient and the healthcare personnel. Telehealth has proven to be useful for rural clinicians dealing with an acute situation and it can include remote specialist reading of an ECG trace, remote prescribing, and instruction on and observation of treatment. This requires adequate investment in emergency telehealth services. Telehealth has been reported to be a satisfactory tool for healthcare personnel and, to support nurses, providing an opportunity for healthcare personnel to learn [123]. Barriers to the uptake of telehealth for ongoing management largely reflect funding and reimbursement arrangements [124], although telehealth offers the potential for more ready access to specialist input. Over time, there will be an improvement of services, as a result of ongoing efforts at quality improvement and sometimes on the back of investigations of failure of care, which result in review and improvements. Technology has also allowed for new opportunities for the way in which care can be delivered, particularly with remote sensing and monitoring. For those with the motivation and means for self-management, there are many opportunities to learn more about their condition and be in control, particularly when the aspects of their care can be accessed in their own home. However, whether these developments reduce the disparities between outcomes for those based rurally and those in urban areas remains to be seen.

## 8. Conclusions

Disparities in CVD incidence and outcomes remain among populations living in rural areas. This review has summarised evidence regarding many of the issues that impact on clinicians managing patients with acute MI in rural areas and in optimising their emergency and follow up care. Rural clinicians need to act quickly to achieve optimal patient outcomes, given the urgency in acute management of ACS. New technologies and quality improvement approaches can enable more access to rapid diagnosis as well as specialist input and care. Approaches for reducing the burden of CVD in rural areas must embrace primary prevention strategies, as well as improvements in diagnosis and management of acute disease and post-discharge care. Linkages between primary and hospital based care are likely to be particularly important in a rural context. It is clear that the issues are complex and they require ongoing support from governments, policy makers, and health professionals with consumer and carer input.
